# Marital Adjustment as a Mediator Between Emotional Suppression and Self-Compassion in Women Aged 35+ Undergoing In Vitro Fertilization-Embryo Transfer: A Cross-Sectional Observational Study

**DOI:** 10.1155/da/2100969

**Published:** 2025-07-15

**Authors:** Mingxiang Zheng, Hengxu Wang, Chaofeng Li, Yan Ouyang, Liyuan Yan, Fei Gong, Li Li, Xihong Li

**Affiliations:** ^1^NHC Key Laboratory of Human Stem Cell and Reproductive Engineering, School of Basic Medical Sciences, Central South University, Changsha City 410078, China; ^2^Imaging Department, Clinical Research Centre for Reproduction and Genetics in Hunan Province, Reproductive and Genetic Hospital of CITIC-Xiangya, Changsha City 410078, China; ^3^College of Nursing, Changsha Medical University, Changsha City 410219, China; ^4^Pediatric Department, Hunan Provincial Key Laboratory of Regional Hereditary Birth Defects Prevention and Control, Changsha Hospital for Maternal and Child Health Care Affiliated With Hunan Normal University, Changsha City 410007, China; ^5^Reproductive Centre, Clinical Research Centre for Reproduction and Genetics in Hunan Province, Reproductive and Genetic Hospital of CITIC-Xiangya, Changsha City 410078, China; ^6^Nursing Department, Clinical Research Centre for Reproduction and Genetics in Hunan Province, Reproductive and Genetic Hospital of CITIC-Xiangya, Changsha City 410078, China; ^7^Xiangya School of Nursing, Central South University, Changsha City 410083, China

**Keywords:** advanced maternal age, emotional suppression, IVF-ET, marital satisfaction, self-compassion

## Abstract

**Objective:** This study aimed to investigate the pathways of emotional suppression among women aged 35 years and older undergoing in vitro fertilization-embryo transfer (IVF-ET) and to provide a theoretical basis for developing personalized programs to reduce emotional suppression.

**Methods:** A convenience sample of 225 women aged ≥35 years undergoing IVF-ET at a reproductive centre between May 1st and September 30th, 2023, was selected. The participants completed a self-designed general information questionnaire, the Emotional Inhibition Scale (EIS), the Locke-Wallace Marriage Adjustment Test (MAT), and the Self-Compassion Scale (SCS). Data were analysed using SPSS 27.0 and AMOS 24.0. Normality and homogeneity of variance were assessed, with normally distributed data described as mean ± standard deviation (SD). Pearson correlation, structural equation modeling (SEM), and bootstrap resampling (5000 iterations, 95% confidence intervals [CIs]) were employed to test the mediation effects, with a bootstrap analysis used to assess the stability and significance of indirect effects through repeated sampling.

**Results:** The cohort comprised predominantly Han Chinese (82.2%), first-married (60.9%), urban-dwelling (60.4%) women with secondary infertility (68.0%). Over 40.0% of the total held a tertiary educational level, and 67.1% reported per capita monthly income of the family (3001–10,000 RMB). The mean emotional suppression score was 28.65 ± 6.74. Emotional suppression was negatively correlated with marital adjustment (*r* = −0.442, *p*  < 0.01) and self-compassion (*r* = −0.393, *p*  < 0.01). SEM with bootstrap validation demonstrated that marital adjustment mediated the relationship between emotional suppression and self-compassion (mediating effect proportion: 24.89%, 95% CI: −0.115 to −0.027).

**Conclusion:** Marital adjustment mediates emotional suppression and self-compassion in women aged ≥35 years undergoing IVF-ET. Fostering positive marital dynamics may alleviate emotional suppression and enhance self-compassion, promoting psychological resilience during treatment. These findings support targeted interventions to improve patient satisfaction and treatment success rates.

## 1. Introduction

Infertility, impacting 1 in 6 adults globally [1], has emerged as a critical public health challenge. China exemplifies this crisis, with infertility rates escalating in 2023 [2]. This surge intersects with profound demographic shifts, while women increasingly delay childbearing beyond age 35 to pursue education and careers, policy changes, such as China's universal two-child policy reveal that 60% of eligible women were over 35 years old, and half exceeded 40 [3]. Although Advanced maternal age (AMA) introduces compounded biological risks, including diminished ovarian reserve, reduced oocyte quality, and impaired endometrial receptivity, all contributing to lower embryo viability and implantation success [4]. Concurrently, AMA infertility care embodies a psychosocial crisis, as women over 35 constitute over 70% of China's assisted reproductive technology (ART) patients [5], facing elevated risks of postpartum depression linked to in vitro fertilization (IVF) conception and repeated treatment cycles [6].

Central to this crisis is emotional suppression, the behavior of individuals consciously inhibiting and hiding the expression of negative emotions [7]. Specifically, patients often conceal their inner pain and emotions under a seemingly calm exterior, maintain a highly rational attitude to control emotional expression, and consciously repress negative emotions. This coping mechanism prevents patients' negative emotions from being released appropriately, which further intensifies these emotions and potentially leads to severe anxiety and depression [8]. Long-term emotional suppression not only fails to effectively alleviate patients' inner pain but may also exacerbate negative emotions such as anxiety and depression, which may adversely affect the effectiveness of IVF-embryo transfer (IVF-ET) treatment and pregnancy outcomes [9]. Foreign studies indicate that older individuals are more likely to adopt emotional suppression strategies than younger women are [10].

The literature identifies critical gaps in addressing this multifaceted challenge. First, an analysis of adaptive self-management traits and psychopathology in infertile women demonstrates that emotional regulation strategies such as self-compassion and the nonsuppression of emotions buffer against depression and psychological distress, particularly in primary infertility [11]. However, the research has predominantly treated infertility as a homogeneous condition, overlooking the unique challenges faced by women who have a greater than 35.38% annual miscarriage risk increase post-35 [12] and workplace inflexibility during ART (reported by 70% of employed women [13]). Second, previous research has focused on anger suppression, neglecting other dimensions of emotional suppression (timidity, verbal inhibition, self-control, and emotional camouflage) [14]. Third, while marital adjustment buffers treatment distress through dyadic coping [15], its mediating role between emotional suppression and self-compassion, a protective intrapersonal resource involving self-kindness during adversity [16, 17], remains unexplored in AMA populations. This gap persists despite evidence that marital dynamics uniquely influence emotional regulation in ART [18].

**Figure 1:**
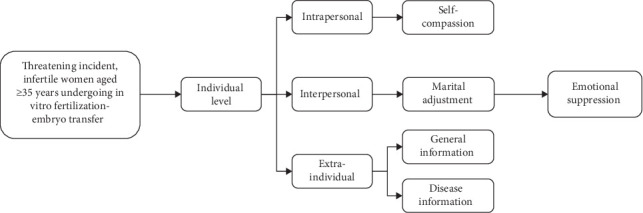
Theoretical framework diagram of this study.

Using the Newman Health Systems Model [19] as a guide, this study proposes that emotional suppression is a dynamic outcome of the interplay of stressors such as inadequate self-compassion (individual level), poor marital adjustment (interpersonal level), and age-related urgency to procreate (environmental level). Although previous studies have shown that self-compassion reduces emotional suppression in patients with chronic diseases [16, 17] and that marital discord exacerbates infertility stress [20, 21], no study has integrated these factors to explain emotional suppression in older women undergoing IVF-ET. The theoretical framework of this study is detailed in Figure 1.

Therefore, the present study aimed to fill three research gaps: first, to focus on the senior maternal population and explore their unique biological and social vulnerabilities; second, to expand other dimensions of emotional inhibition to include timidity, verbal inhibition, self-control, and emotional camouflage; and third, to validate the interaction between self-compassion (individual level) and marital adjustment (interpersonal level) in the context of age-related fertility stress (environmental level) mechanism. By applying the Newman model, the results will shed light on how multiple levels of stressors work together to drive emotional inhibition, with the aim of improving mental health and ART treatment outcomes.

## 2. Materials and Methods

### 2.1. Study Objects

This study employed a convenience sampling method and selected a reproductive centre in Changsha City. Female patients who met the inclusion and exclusion criteria and underwent IVF-ET treatment at this reproductive centre between May 1st, 2023, and September 30th, 2023, were selected as study subjects.

Inclusion criteria:

1. Diagnosed with infertility according to standard criteria [1].
2. Met the indications for IVF-ET-assisted reproduction and signed up for the treatment cycle.
3. Age ≥35 years.
4. Able to independently read, comprehend, and complete self-administered questionnaires in Chinese without assistance (e.g., due to deafness, blindness, or cognitive impairments affecting communication).
5. Verbally confirmed understanding of the study's purpose, procedures, and voluntary nature during the informed consent process, and voluntarily signed the consent form.

Exclusion criteria:

1. History of or current mental illness.
2. Concurrent with other severe physical illnesses.

The sample size calculation formula used was *n* = 1 + *m* + *m*^2^ (1/*R*^2^ − 1) [22]. The number of independent variables was *m* = 30 (19 general data points, 4 emotional suppression variables, 1 marital adjustment variable, and 6 self-compassion variables). For a two-sided test with *α* = 0.05, *ψ* = 1.96. Using the same survey tools and questionnaires as those used in the formal investigation, a linear regression analysis of factors influencing emotional suppression among women 35 years and older undergoing IVF-ET from a pilot survey yielded *R*^2^ = 0.414. Therefore, *n* = 1 + 30 + 30 × 1.96^2^ (1/0.414−1) = 194. Considering a 15% invalid questionnaire rate, the minimum sample size was determined to be 223 cases. Ultimately, 225 patients were included in this study. This study obtained approval from the Ethics Committee of our hospital (Approval Number: LL-SC-2023-022).

### 2.2. Research Methods

#### 2.2.1. Self-Compiled General Information Questionnaire

The questionnaire was developed by the researcher after a review of the literature and medical records. The questionnaire included age, ethnicity, marital status, place of residence, education level, occupation, whether the participant was an only child, the presence of children, personality type, sleep quality, family life type, monthly per capita family income, duration of infertility, type of infertility, infertility factors, infertility-related expenses, history of miscarriage, history of ART, and current assisted reproduction cycle.

#### 2.2.2. Emotional Inhibition Scale (EIS)

Developed by Kellner [23] and subsequently translated into Chinese by Liping et al. [24], the EIS is used to measure an individual's level of emotional inhibition. The scale consists of 14 items divided into four dimensions: timidity (4 items), verbal inhibition (3 items), self-control (4 items), and emotional faux pas (3 items). It uses a 5-point rating scale ranging from 0 (never) to 4 (always), with items 2, 4, 5, and 14 scored in reverse. The total score ranges from 0 to 56, with higher scores indicating higher levels of emotional inhibition. The Cronbach's *α* coefficient of this scale was 0.72, and the test‒retest reliability was 0.855, indicating good reliability and validity. In our sample of women undergoing IVF-ET, the scale demonstrated acceptable internal consistency (Cronbach's *α* = 0.70) [25].

#### 2.2.3. Locke-Wallace Marriage Adjustment Test (MAT)

Developed by Locke and Wallace [26] and translated into Chinese by Wang et al. [27], the MAT is used to assess the intimacy and marital happiness of married couples. It consists of 15 items with a total score that is the sum of all items. The total score ranges from 2 to 158, with higher scores indicating better intimacy and marital quality. A score below 100 indicates marital discord, whereas a score of 100 or above indicates good marital adjustment. The Cronbach's *α* coefficient of this scale was 0.70 [28], indicating good reliability and validity. In our sample of women undergoing IVF-ET, the scale demonstrated acceptable internal consistency (Cronbach's *α* = 0.72) [25].

#### 2.2.4. Self-Compassion Scale (SCS)

Developed by Neff [29] and translated into Chinese by Chen Jian et al. [30], the SCS is used to assess an individual's level of self-compassion. The scale consists of 26 items divided into six dimensions: self-kindness (5 items), self-judgement (5 items), common humanity (4 items), isolation (4 items), mindfulness (4 items), and overidentification (4 items). It uses a 5-point rating scale ranging from 1 (never) to 5 (almost always), with items in the self-judgement, isolation, and overidentification dimensions scored in reverse. The total score ranges from 26 to 130, with higher scores indicating higher levels of self-compassion. The Cronbach's *α* coefficient of this scale was 0.84 and the test‒retest reliability coefficient after 2 weeks was 0.89, indicating good reliability and validity. In our sample of women undergoing IVF-ET, the scale demonstrated acceptable internal consistency (Cronbach's *α* = 0.82) [25].

### 2.3. Data Collection Method

This study adopted an on-site questionnaire survey method. With the approval of hospital leadership, eligible participants were surveyed in the contract talking room of a reproductive specialist hospital in Changsha, Hunan Province. All researchers received unified training to ensure that they understood the research details and communication skills. Before the investigation, the participants were clearly informed of the purpose, content, and expected duration of the research. After informed consent was obtained, questionnaires were distributed with unified instructions. The questionnaires were collected immediately upon completion and checked for completeness to ensure that no data were missing. Each questionnaire took approximately 20 min to complete. A total of 230 questionnaires were distributed, and 5 invalid questionnaires were excluded due to incomplete answers (*n* = 2), identical answers (*n* = 1), contradictory answers (*n* = 1), and obvious errors (*n* = 1), leaving 225 valid questionnaires for the final analysis. The final analysis included 225 women aged ≥35 years, with the following ART distributions: IVF (*n* = 23, 10.2%), intracytoplasmic sperm injection (ICSI) (*n* = 9, 4.0%), preimplantation genetic testing (PGT) (*n* = 57, 25.3%), and IVF/ICSI (*n* = 136, 60.4%).

### 2.4. Statistical Methods

SPSS 27.0 and AMOS 24.0 were employed for data analysis and processing. The quantitative data were tested for normality and homogeneity of variance. If the data met the criteria for normal distribution and homogeneity of variance, they were described using the mean ± standard deviation (SD; *x* ± *s*). Otherwise, the median and quartiles (*M* [P25, P75]) were used for description. Qualitative data are presented as frequencies and percentages (%). Independent-sample *t* tests and one-way ANOVA were conducted for a univariate analysis of variance. A Pearson correlation analysis was performed. A multiple linear regression analysis was adopted to investigate the factors that influenced emotional inhibition among women 35 years and older undergoing IVF-ET. Multicollinearity diagnostics for predictors were performed using the variance inflation factor (VIF) method. A higher VIF value indicates more severe multicollinearity [31]. Multicollinearity is generally accepted to occur among independent variables when the VIF exceeds 10 [32]. AMOS 24.0 software was used for structural equation modeling (SEM) to verify the variable relationships and mediating effects [33]. The bootstrap method was used to validate the mediation effect of the model, with 5000 resamples performed to calculate the 95% confidence intervals (CIs) for each effect value. The maximum likelihood ratio method was employed to revise and fit the model, thereby validating the hypothesis. The significance level was set at *α* = 0.05, and a *p* value < 0.05 was considered statistically significant.

## 3. Results

### 3.1. Comparison of Emotional Inhibition Scores Among Women 35 Years and Older Undergoing IVF-ET With Different Characteristics

Among the 225 patients in this group, the cohort comprised predominantly Han Chinese (82.2%), first-married (60.9%), urban-dwelling (60.4%) women with secondary infertility (68.0%). Over 40.0% of the total held a tertiary educational level, and 67.1% reported per capita monthly income of the family (3001–10,000 RMB). Women 35 years and older who were remarried, had secondary infertility, were infertile due to female factors, lived in rural areas, had a lower level of education, had lower per capita monthly income, were not only children, had a longer duration of infertility, and incurred higher infertility-related expenses scored higher on emotional inhibition. The differences were statistically significant (all *p*  < 0.05). The specific results are shown in Table 1.

### 3.2. Scores and Correlation Analysis of Emotional Inhibition, Marital Adjustment, and Self-Compassion Among Women 35 Years and Older Undergoing IVF-ET

In this study, the emotional inhibition score for women 35 years and older undergoing IVF-ET was 28.65 ± 6.74, the marital adjustment score was 104.52 ± 24.05, and the self-compassion score was 89.94 ± 11.78. A Pearson correlation analysis revealed that emotional inhibition was negatively correlated with marital adjustment (*r* = −0.442, *p*  < 0.01) and self-compassion (*r* = −0.393, *p*  < 0.01), whereas marital adjustment was positively correlated with self-compassion (*r* = 0.397, *p*  < 0.01) among women 35 years and older undergoing IVF-ET. The specific results are shown in Table 2.

### 3.3. Multivariate Analysis of Emotional Inhibition Among Women 35 Years and Older Undergoing IVF-ET

Taking emotional inhibition as the dependent variable and statistically significant variables from the univariate analysis and correlation analysis as independent variables, multiple linear regression analysis was conducted. The results showed that infertility factors, marital adjustment, and self-compassion entered the regression equation and collectively explained 32.8% of the total variation.  Table 3 for details.

### 3.4. Construction and Validation of the Structural Equation Model

**Figure 2:**
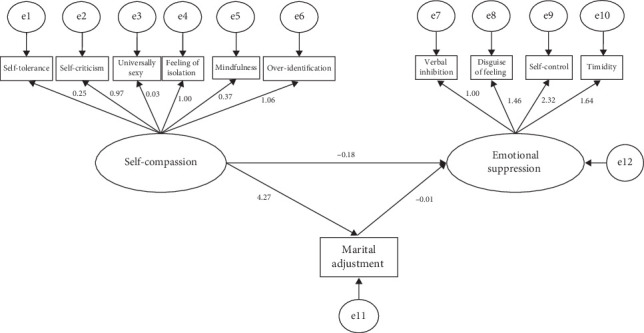
The mediating effect of marital adjustment on the relationship between self-compassion and emotional suppression.

The VIF obtained from the multicollinearity test in this study was <10, indicating that there were no multicollinearity issues among the variables. In the mediation model of this study, to prevent potential confounding effects from other variables and enhance the statistical power of the tests, the decision was made to include infertility-related factors in the covariates module based on the results of the multivariate analysis. These variables were controlled for during the bootstrapping procedures. A structural equation model was constructed with self-compassion as the independent variable, emotional inhibition as the dependent variable, and marital adjustment as the mediator. The initial model was fitted using the maximum likelihood ratio method. The final results were *x*^2^/df = 1.748, RMSEA = 0.058, TLI = 0.933, IFI = 0.962, NFI = 0.915, and CFI = 0.961, which all reached ideal values and indicated a good fit of the constructed structural equation model. The path diagram of the structural equation model is shown in Figure 2.

### 3.5. Mediating Role of Marital Adjustment in the Relationship Between Emotional Inhibition and Self-Compassion Among Women 35 Years and Older Undergoing IVF-ET

The mediating effect of marital adjustment on the relationship between emotional inhibition and self-compassion among women 35 years and older undergoing IVF-ET was tested using the bootstrap method with 5000 resamples and a 95% CI. The results, shown in Table 4, indicated that the 95% CI did not include 0, suggesting that marital adjustment had a mediating role. The effect size was −0.059, which accounted for 24.89% of the total effect.

## 4. Discussion

This study elucidates the complex interplay among emotional inhibition, marital adjustment, and self-compassion in women aged ≥35 undergoing IVF-ET. Guided by Neuman's Systems Model, SEM revealed that marital adjustment partially mediates the relationship between self-compassion and emotional inhibition (24.89% of total effect), highlighting the dual pathways through which psychological and relational resources mitigate distress during infertility treatment.

A univariate analysis identified higher emotional inhibition levels among remarried women, those with secondary infertility, prolonged infertility duration, rural residency, lower education/income, elevated treatment costs, and non-only-child families, consistent with prior studies [34]. In multichild families, intensified familial expectations to “continue the lineage,” particularly in rural areas influenced by traditional pronatalist norms (e.g., “childlessness as unfilial”), coupled with geographic barriers to mental health services, exacerbate stigma and isolation [35–37]. Prolonged infertility erodes self-efficacy and exacerbates financial despair, particularly for low-income families facing prohibitive ART costs (30,000–50,000 RMB/cycle), a key driver of treatment discontinuation [38]. Limited health literacy among less-educated individuals perpetuates reliance on nonscientific treatments and delays evidence-based care [39]. Women with secondary infertility experience heightened frustration from prior reproductive success contrast, while remarried patients face compounded pressures from blended family dynamics and societal scrutiny of fertility competence.

The cohort's mean emotional inhibition score (28.65 ± 6.74) was higher than that reported in a general Italian population sample (27.21 ± 6.65) of adults aged ≥18 years with no specified recruitment period [40], but lower than that of postpartum women in China (30.48 ± 6.46; women aged ≥20 years assessed 1 week postdelivery at a tertiary hospital, 2022) [41]. This gradient reflects distinct stressors: IVF-ET patients endure prolonged uncertainty and age-related fertility stigma [42], whereas postpartum stress is acute but socially validated. Notably, women with female-factor infertility exhibited the highest inhibition levels, aligning with collectivist cultural paradigms equating womanhood with motherhood [43]. A multivariate analysis confirmed female-attributed infertility as the primary predictor of emotional suppression in this population [44]. In traditional Chinese society, women's social value remains inextricably linked to reproductive capacity, fostering self-blame and perceived bodily inadequacy. This situation necessitates targeted societal education to dismantle pronatalist stereotypes and cultivate inclusive support systems.

The mean score of marital adjustment in this study (104.52 ± 24.05) indicates overall good marital adjustment. Significant negative correlations emerged between marital adjustment and emotional suppression (*r* = −0.442, *p*  < 0.01) [45], underscoring the protective role of spousal support. Effective marital adjustment creates a secure environment for emotional expression, facilitating collaborative coping strategies under fertility stress. Conversely, poor adjustment may trigger maladaptive inhibition cycles to preserve marital stability, potentially compromising treatment adherence [46–48]. Clinical implementation of couples' counseling to enhance problem-solving and empathic communication is strongly recommended. Similarly, self-compassion demonstrated a negative association with emotional suppression (*r* = −0.393, *p*  < 0.01) [49], mediated partially through marital adjustment. High self-compassion individuals employ self-kindness to mitigate self-criticism and common humanity perspectives to contextualize infertility challenges [50], countering “infertility-as-defect” cognitive distortions. Integrating self-compassion training (mindfulness or self-compassion journaling) into psychosocial interventions can establish adaptive emotional regulation mechanisms.

The 24.89% mediation effect of marital adjustment reveals dual pathways: self-compassion directly buffers psychological stress while indirectly reducing emotional internalization through enhanced relational dynamics. For older infertile women facing compounded sociocultural pressures, marital quality serves as a critical modulator. Self-compassion training reduces self-judgment and bolsters emotional resilience [51]; thus, self-compassion is particularly beneficial for populations, such as PCOS patients [52]. Concurrently, self-compassion fosters spousal empathy and supportive communication, partners attuned to conflict dynamics respond with compassion rather than reactivity [53], thereby mitigating stigma-driven inhibition. Evidence-based dyadic interventions improve marital satisfaction by addressing stress, communication, and conflict resolution [54]. Positive marital interactions disrupt “stress-silencing” cycles through shared decision-making and practical support (e.g., financial burden-sharing) [55], aligning with mindfulness interventions that enhance relational empathy [56]. Reproductive health providers should prioritize self-compassion training and couples counseling to disrupt maladaptive stress cycles, thereby reducing emotional inhibition and improving treatment outcomes for older infertile women.

This study has notable methodological constraints. First, the inclusion criteria excluded individuals with sensory/communicative impairments (e.g., deafness or blindness), potentially limiting the generalizability to women aged ≥35. Second, the exclusion criteria (psychiatric history, current mental disorders, or severe somatic diseases) introduced selection bias by omitting high-risk subgroups, thus reducing applicability to vulnerable populations. Third, convenience sampling from a single institution restricted socioeconomic and geographical diversity. Fourth, the cross-sectional design precludes causal inference between psychological states and IVF-ET treatment processes, as temporal relationships remain undetermined. Fifth, our analysis did not fully account for sociocultural confounders inherent to the study population. Pronatalist norms prevalent in collectivist societies may systematically influence participants' emotional regulation patterns and treatment expectations, potentially confounding the observed psychological outcomes. Additionally, unmeasured relational dynamics, including spousal support granularity, extended family pressures, or culturally mediated stigma surrounding infertility, could introduce residual bias in the emotional inhibition pathways. To address these limitations, future investigations should prioritize longitudinal designs to track dynamic psychological and physiological changes (e.g., cortisol levels) across treatment cycles, while integrating spousal support variables to contextualize relational dynamics. Multicentre collaborations utilizing stratified sampling could enhance demographic representativeness across regions, socioeconomic strata, and special populations with communication needs (e.g., incorporating adaptive strategies, such as sign language support). Methodological triangulation, combining clinical assessments, biomarker data, and qualitative insights, should supplement self-reports to minimize bias. Transparent justification for variable selection (e.g., rationale for excluding psychiatric comorbidities) is also warranted. Future studies should incorporate validated cultural metrics (e.g., regional fertility norms and gender role attitudes) and employ dyadic assessment frameworks to capture these multidimensional influences.

## 5. Conclusions

In conclusion, this study elucidates the dual pathways through which marital adjustment mediates the interplay between self-compassion and emotional suppression in women aged ≥35 undergoing IVF-ET. The findings underscore that emotional suppression is not merely an outcome but a dynamic process moderated by relational dynamics. Specifically, higher self-compassion directly alleviates psychological distress while indirectly reducing emotional inhibition via improved marital adjustment (24.89% mediation effect). These insights call for interventions that simultaneously target intrapersonal resilience (e.g., self-compassion training) and relational resources (e.g., couples' conflict resolution) to disrupt maladaptive stress cycles. By addressing both pathways, healthcare providers can mitigate the unique sociocultural and financial pressures faced by older, infertile women, thereby enhancing treatment adherence and psychological well-being.

## Figures and Tables

**Table 1:** Comparison of Emotional Inhibition Scale scores among women 35 years and older with different characteristics undergoing IVF-ET (*n* = 225).

Variable	Participants, *n* (%)	EIS score, mean (SD)	*t*/*F* value	*p*-Value
Ethnicity	—	—	−1.820	0.074
Han	185 (82.22)	28.25 ± 6.60	—	—
Ethnic minorities	40 (17.78)	30.50 ± 7.18	—	—
Marital status	—	—	−3.211	0.002
First marriage	137 (60.89)	27.56 ± 7.05	—	—
Remarriage	88 (39.11)	30.35 ± 5.87	—	—
Family life type	—	—	1.628	0.184
Living with spouse only	113 (50.22)	28.63 ± 6.36	—	—
Living with parents	56 (24.89)	28.30 ± 7.11	—	—
Living with spouse and children	48 (21.33)	29.83 ± 6.62	—	—
Living separately	8 (3.56)	24.38 ± 9.27	—	—
Infertility type	—	—	−2.332	0.021
Primary infertility	72 (32.00)	27.13 ± 6.81	—	—
Secondary infertility	153 (68.00)	29.37 ± 6.61	—	—
Infertility factor	—	—	38.761	<0.001
Female factor	113 (50.22)	32.24 ± 4.41	—	—
Male factor	35 (15.56)	21.63 ± 6.56	—	—
Both factors	46 (20.44)	26.46 ± 5.77	—	—
Unknown cause	31 (13.78)	26.77 ± 7.16	—	—
Place of residence	—	—	−4.210	<0.001
Urban	136 (60.44)	27.18 ± 7.03	—	—
Rural	89 (39.56)	30.91 ± 5.60	—	—
Education level	—	—	5.566	0.001
Junior high school and below	67 (29.78)	31.09 ± 5.53	—	—
High school or technical secondary school	61 (27.11)	28.77 ± 6.96	—	—
College or undergraduate	83 (36.89)	27.07 ± 7.07	—	—
Master's degree and above	14 (6.22)	25.86 ± 5.88	—	—
Occupation	—	—	1.922	0.092
None	61 (27.11)	29.62 ± 7.00	—	—
Self-employed	34 (15.11)	29.62 ± 6.11	—	—
Worker	7 (3.11)	29.29 ± 6.65	—	—
Public institution employe	41 (18.22)	26.34 ± 7.06	—	—
Farmer	16 (7.11)	31.06 ± 5.56	—	—
Clerk	66 (29.33)	28.05 ± 6.65	—	—
Monthly per capita income	—	—	5.407	0.001
≤3000	43 (19.11)	30.56 ± 5.84	—	—
3001–5000	85 (37.78)	29.65 ± 6.58	—	—
5001–10,000	66 (29.33)	27.85 ± 6.72	—	—
>10,000	31 (13.78)	25.00 ± 7.03	—	—
Personality type	—	—	2.476	0.086
Introverted	38 (16.89)	30.66 ± 5.15	—	—
Extroverted	58 (25.78)	27.57 ± 7.21	—	—
Ambiverted	129 (57.33)	28.55 ± 6.86	—	—
Sleep quality	—	—	2.645	0.073
Poor	22 (9.78)	30.41 ± 6.14	—	—
Average	144 (64.00)	29.03 ± 6.76	—	—
Good	59 (26.22)	27.07 ± 6.73	—	—
History of miscarriage	—	—	1.714	0.165
None	89 (39.56)	27.46 ± 6.65	—	—
1 Time	59 (26.22)	29.63 ± 6.96	—	—
2 Times	41 (18.22)	28.83 ± 6.34	—	—
3 Times or more	36 (16.00)	29.81 ± 6.87	—	—
Presence of children	—	—	1.253	0.211
Yes	107 (47.56)	29.24 ± 6.57	—	—
No	118 (52.44)	28.12 ± 6.88	—	—
Only child status	—	—	−2.569	0.011
Yes	40 (17.78)	26.20 ± 8.14	—	—
No	185 (82.22)	29.18 ± 6.30	—	—
Duration of infertility	—	—	3.809	0.024
<3 years	63 (28.00)	26.70 ± 6.75	—	—
3–5 years	55 (24.44)	29.62 ± 6.78	—	—
>5 years	107 (47.56)	29.31 ± 6.55	—	—
Cost of infertility treatment	—	—	4.368	0.005
<10,000	26 (11.56)	24.54 ± 7.09	—	—
10,000–30,000	65 (28.89)	28.72 ± 6.77	—	—
30,001–50,000	30 (13.33)	28.27 ± 5.15	—	—
>50,000	104 (46.22)	29.75 ± 6.73	—	—
History of assisted reproduction	—	—	0.155	0.877
Yes	100 (44.44)	28.73 ± 6.07	—	—
No	125 (55.56)	28.59 ± 7.26	—	—
Current assisted reproduction cycle	—	—	0.863	0.461
1st cycle	132 (58.67)	28.87 ± 7.27	—	—
2nd cycle	53 (23.56)	28.96 ± 5.81	—	—
3rd–4th cycle	34 (15.11)	27.03 ± 6.13	—	—
5th cycle or more	6 (2.67)	30.33 ± 5.61	—	—

**Table 2:** Correlation analysis of emotional inhibition, marital adjustment, and self-compassion in women 35 years and older undergoing IVF-ET.

Variable	Score	1	2	3	4	5	6	7	8	9	10	11	12	13
1	7.67 ± 2.41	1	—	—	—	—	—	—	—	—	—	—	—	—
2	9.02 ± 2.35	0.294*⁣*^*∗∗*^	1	—	—	—	—	—	—	—	—	—	—	—
3	7.22 ± 3.12	0.037	0.298*⁣*^*∗∗*^	1	—	—	—	—	—	—	—	—	—	—
4	4.74 ± 2.30	0.226*⁣*^*∗∗*^	0.405*⁣*^*∗∗*^	0.273*⁣*^*∗∗*^	1	—	—	—	—	—	—	—	—	—
5	28.65 ± 6.74	0.554*⁣*^*∗∗*^	0.730*⁣*^*∗∗*^	0.673*⁣*^*∗∗*^	0.690*⁣*^*∗∗*^	1	—	—	—	—	—	—	—	—
6	104.52 ± 24.05	−0.303*⁣*^*∗∗*^	−0.357*⁣*^*∗∗*^	−0.173*⁣*^*∗∗*^	−0.378*⁣*^*∗∗*^	−0.442*⁣*^*∗∗*^	1	—	—	—	—	—	—	—
7	16.73 ± 3.75	−0.371*⁣*^*∗∗*^	−0.079	0.133*⁣*^*∗*^	−0.170*⁣*^*∗*^	−0.157*⁣*^*∗*^	0.205*⁣*^*∗∗*^	1	—	—	—	—	—	—
8	18.68 ± 3.10	−0.221*⁣*^*∗∗*^	−0.335*⁣*^*∗∗*^	−0.170*⁣*^*∗*^	−0.454*⁣*^*∗∗*^	−0.430*⁣*^*∗∗*^	0.280*⁣*^*∗∗*^	0.08	1	—	—	—	—	—
9	12.25 ± 3.06	−0.310*⁣*^*∗∗*^	−0.067	−0.007	−0.143*⁣*^*∗*^	−0.186*⁣*^*∗∗*^	0.158*⁣*^*∗*^	0.513*⁣*^*∗∗*^	−0.026	1	—	—	—	—
10	14.60 ± 2.95	−0.152*⁣*^*∗*^	−0.289*⁣*^*∗∗*^	0.037	−0.375*⁣*^*∗∗*^	−0.266*⁣*^*∗∗*^	0.317*⁣*^*∗∗*^	0.042	0.535*⁣*^*∗∗*^	0.118	1	—	—	—
11	13.15 ± 3.11	−0.359*⁣*^*∗∗*^	−0.13	0.048	−0.212*⁣*^*∗∗*^	−0.224*⁣*^*∗∗*^	0.252*⁣*^*∗∗*^	0.638*⁣*^*∗∗*^	0.113	0.501*⁣*^*∗∗*^	0.148*⁣*^*∗*^	1	—	—
12	14.53 ± 2.96	−0.07	−0.224*⁣*^*∗∗*^	0.023	−0.380*⁣*^*∗∗*^	−0.222*⁣*^*∗∗*^	0.283*⁣*^*∗∗*^	−0.034	0.554*⁣*^*∗∗*^	−0.012	0.604*⁣*^*∗∗*^	0.199*⁣*^*∗∗*^	1	—
13	89.94 ± 11.78	−0.407*⁣*^*∗∗*^	−0.294*⁣*^*∗∗*^	0.023	−0.456*⁣*^*∗∗*^	−0.393*⁣*^*∗∗*^	0.397*⁣*^*∗∗*^	0.643*⁣*^*∗∗*^	0.585*⁣*^*∗∗*^	0.575*⁣*^*∗∗*^	0.626*⁣*^*∗∗*^	0.714*⁣*^*∗∗*^	0.587*⁣*^*∗∗*^	1

*Note:* 1, verbal inhibition; 2, emotional pretense; 3, self-control; 4, timidity; 5, total score of emotional inhibition; 6, marital adjustment; 7, self-kindness; 8, self-criticism; 9, sense of universal humanity; 10, sense of isolation; 11, mindfulness; 12, over-identification; 13, total score of self-compassion.

*⁣* ^*∗*^ *p* < 0.05.

*⁣* ^*∗∗*^ *p* < 0.01.

**Table 3:** Multiple linear regression analysis of emotional inhibition in women 35 years and older undergoing IVF-ET.

Independent variables	*B*	SE	Beta	*t*	*p*
Constant	40.102	4.852	—	8.266	<0.001
Infertility factors (with female factors as the reference)	−1.194	0.371	−0.199	−3.217	0.001
SCS	−0.088	0.037	−0.153	−2.382	0.018
MAT	−0.084	0.017	−0.301	−4.869	<0.001

*Note:* Marital status: (first marriage [reference] = 1, remarriage = 2); infertility type: (primary infertility [reference] = 1, secondary infertility = 2); infertility factors: (dummy-coded with female factor as reference; *X*_1_ [male factor] = [1, 0, 0]; *X*_2_ [both factors] = [0, 1, 0]; *X*₃ [unknown cause] = [0, 0, 1]); place of residence: (urban [reference] = 1; rural = 2); only child status: (yes [reference] = 1; no = 2). Adjusted *R*^2^ = 0.328, *F* = 10.934, and *p* < 0.001.

Abbreviations: MAT, Locke-Wallace Marital Adjustment Test; SCS, Self-Compassion Scale.

**Table 4:** Bootstrap mediating effect test results.

Effect	Value	SE	95% CI	*p*	Proportion
Direct effect	−0.178	0.044	−0.283 to –0.106	<0.001	75.11%
Mediated effect	−0.059	0.022	−0.115 to –0.027	<0.001	24.89%
Total effect	−0.237	0.053	−0.359 to –0.147	<0.001	100.00%

## Data Availability

The data that support the findings of this study are available from the corresponding author upon reasonable request.

## Nomenclature

IVF-ET: In vitro fertilization-embryo transfer
MAT: Marital adjustment test
ART: Assisted reproductive technology
EIS: Emotional inhibition scale
SCS: Self-compassion scale
SEM: Structural equation modeling
CI: Confidence interval
VIF: Variance inflation factor.
