# Correction to “Marital Adjustment as a Mediator Between Emotional Suppression and Self‐Compassion in Women Aged 35+ Undergoing In Vitro Fertilization‐Embryo Transfer: A Cross‐Sectional Observational Study”

**DOI:** 10.1155/da/9868160

**Published:** 2026-02-14

M. Zheng, H. Wang, C. Li, et al., “Marital Adjustment as a Mediator Between Emotional Suppression and Self‐Compassion in Women Aged 35+ Undergoing In Vitro Fertilization‐Embryo Transfer: A Cross‐Sectional Observational Study,” *Depression and Anxiety* (2025): 2025 2100969, https://doi.org/10.1155/da/2100969.

In the article titled, “Marital Adjustment as a Mediator Between Emotional Suppression and Self‐Compassion in Women Aged 35+ Undergoing In Vitro Fertilization‐Embryo Transfer: A Cross‐Sectional Observational Study,” there was an error in Figure 1. The figure should show with the connecting lines that illustrate the conceptual relationships between variables. The correct Figure 1 is shown below:

**Figure 1:**
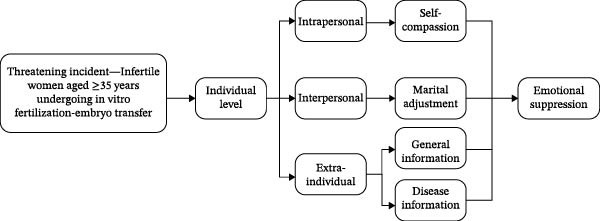
Theoretical framework diagram of this study.

We apologize for this error.

